# Effect of dietary myo-inositol supplementation on the insulin resistance and the prevention of gestational diabetes mellitus: study protocol for a randomized controlled trial

**DOI:** 10.1186/s13063-020-04561-2

**Published:** 2020-07-09

**Authors:** George Asimakopoulos, Vasilios Pergialiotis, Eleni Anastasiou, Panagiotis Antsaklis, Mariana Theodora, Evangelia Vogiatzi, Aggela Kallergi, Michael Sindos, Dimitrios Loutradis, George Daskalakis

**Affiliations:** 1 First Department of Obstetrics and Gynecology, National and Kapodistrian University of Athens, Alexandra Hospital, 80 Vasilissis Sofias Avenue, Athens, Greece; 2 Endocrine Section - Diabetes Centre, Alexandra Hospital, 80 Vasilissis Sofias Avenue, Athens, Greece

**Keywords:** Myo-inositol, Gestational diabetes mellitus, Insulin resistance, RCT

## Abstract

**Background:**

Gestational diabetes mellitus (GDM) is defined as impaired glucose tolerance with onset or first recognition during pregnancy, which is characterized by an increased insulin resistance. Gestational diabetes mellitus is associated with pregnancy-related maternal and fetal morbidity (both antenatal and perinatal).

Myo-inositol has been suggested to improve insulin resistance in women with polycystic ovary syndrome.

The aim of this study is to examine the impact of myo-inositol supplementation during pregnancy on the incidence of gestational diabetes mellitus.

**Methods:**

We will conduct a single-center, open-label, randomized controlled trial. A total of 160 healthy pregnant women with singleton pregnancy at 11–13^+6^ weeks of gestation will be randomly allocated in two groups: intervention group (*N* = 80) and control group (*N* = 80). The intervention group will receive myo-inositol and folic acid (4000 mg myo-inositol and 400 mcg folic acid daily) from 11 to 13^+6^ weeks of gestation until 26–28 weeks of gestation, while the control group will receive folic acid alone (400 mcg folic acid daily) for the same period of time as intervention group. The primary outcome will be gestational diabetes incidence rate at 26–28 weeks of gestation, according to the results of a 75 g oral glucose tolerance test held at 26–28 weeks of gestation. The secondary outcomes will include fasting blood glucose levels, glycated hemoglobin levels, insulin resistance level (evaluated by homeostasis model assessment of insulin resistance and Matsuda Index), and incidence rate of diet-treated gestational diabetes and diabetes requiring insulin therapy at 26–28 weeks of gestation.

**Discussion:**

This trial will provide evidence for the effectiveness of myo-inositol supplementation during pregnancy in reducing the incidence of gestational diabetes mellitus.

**Trial registration:**

ISRCTN registry: ISRCTN16142533. Registered on 9 March 2017.

## Background

Gestational diabetes mellitus (GDM) is defined as impaired glucose tolerance with onset or first recognition during pregnancy, which is characterized by an increased insulin resistance [1, 2]. The prevalence of that complication is rising worldwide [3]. The onset of GDM is associated with several risk factors such as advanced maternal age, increased body mass index (BMI), ethnic background, and family history or previous history of gestational diabetes [2]. Advancing maternal age and increased prevalence of obesity have contributed to the escalation in rates of GDM. Although the reported rates of GDM imply an incidence that reaches 10% of pregnancies, its prevalence is expected to be higher, almost double, according to the newly proposed criteria for diagnosing GDM [4].

Gestational diabetes mellitus is associated with pregnancy-related maternal and fetal morbidity (both antenatal and perinatal). Maternal hyperglycemia is associated with an excessive increase of fetal weight. Perinatal morbidity is increased due to complications such as fetal macrosomia, shoulder dystocia, birth injuries, neonatal hypoglycemia, and respiratory distress syndrome [5]. Maternal morbidity includes, although not limited to, cesarean delivery, hypertensive disorders, preeclampsia, and predisposition to the development of type 2 diabetes (T2D) later in life [6]. The macrosomic newborns are at increased risk for the onset of obesity, hypertension, and diabetes later in life [7]. The increasing prevalence of fetal and maternal complications related to GDM led to increased awareness of this condition worldwide.

Pregnancy is characterized by insulin resistance. This metabolic state seems to be necessary to ensure a continuous supply of nutrients to the fetus even in conditions of famine. Most pregnant women respond to this effect by increasing insulin release and remain euglycemic. On the other hand, women with GDM are unable to counteract insulin resistance. Insulin-sensitizing factors, such as metformin, have been proposed as a potential treatment to reduce the incidence of GDM in high-risk pregnancies. While metformin initially appeared to be a promising intervention for the prevention of the disease, it does not seem to be as efficacious as expected [8]. The combination of intensive monitoring during the pregnancy course and the use of novel insulin regimens renders possible the effective regulation of blood glucose levels and improves the perinatal outcome. However, the incidence of macrosomia does not appear to be significantly reduced, resulting in a large percentage of macrosomic newborns (15–45%) [9].

It is logical to consider that preventive strategies are better than active response following the presentation of a complication. Safe and effective interventions are, therefore, urgently needed in an attempt to lower the incidence of gestational diabetes or at least the incidence of GDM related morbidity. Such strategies will also benefit health care systems as they could significantly decrease the economic burden of the disease in a national and international setting. Previous studies suggested that inositol may significantly decrease insulin levels in women with polycystic ovary syndrome (PCOS) when administered as one of the two isomer forms, myo-inositol [10] or D-chiro-inositol [11].

Inositol phosphoglycan is an intracellular insulin mediator, which is associated with insulin sensitivity in conditions of insulin resistance, such as T2D [12, 13]. Gestational diabetes mellitus is characterized by increased urinary excretion of inositol phosphoglycan, which shows a positive correlation with blood glucose levels [14, 15]. This observation has also been reported in patients with insulin resistance related to PCOS [16], who have been successfully treated with myo-inositol and folic acid [17]. In a previous study, D’Anna et al. suggested that myo-inositol may actually prevent the occurrence of GDM in PCOS women [18]. The insulin resistance of the syndrome seems to be characterized by a defect in tissue availability or utilization of inositol mediator; therefore, the supplementation of that mediator would improve the intracellular effect of insulin [13, 14, 16, 17]. These findings support the hypothesis that inositol administration could decrease insulin resistance related to GDM. Inositol is considered as a dietary supplement rather than a drug and is present in many foods, such as cereals, corn, legumes, and meat. It is synthesized principally by the liver. To date, a handful of randomized trials have assessed the impact of inositol regimens on the incidence of GDM. Of those, two studies that recruited overweight pregnant women observed > 50% reduction in the incidence of GDM [17, 19], and one study that included obese women observed nearly 60% reduction [20]. Women with polycystic ovarian syndrome may also benefit from inositol supplementation during pregnancy as the incidence of GDM seems to be reduced by more than 60% according to the findings of another study [18]. Results, however, seem to be conflicting in cases with a family history of GDM as D’Anna et al. observed a significant effect [13] whereas Farren et al. did not [21].

This randomized, open-label study is designed to evaluate the impact of myo-inositol supplementation from the first trimester of pregnancy on GDM prevalence. Results of this study will provide evidence regarding the value of myo-inositol as a safe intervention for the prevention of GDM.

## Methods/design

A SPIRIT checklist is available online for this manuscript (Additional file 1).

### Aims

We hypothesize that myo-inositol supplementation from 11 to 13^+6^ weeks of gestation until the time of GDM diagnosis (26–28 weeks of gestation) will reduce the incidence of GDM. To test this hypothesis, we will compare an intervention group and a control group. The intervention group will receive myo-inositol and folic acid, while the control group will receive folic acid alone.

### Design and setting

**Fig. 1:**
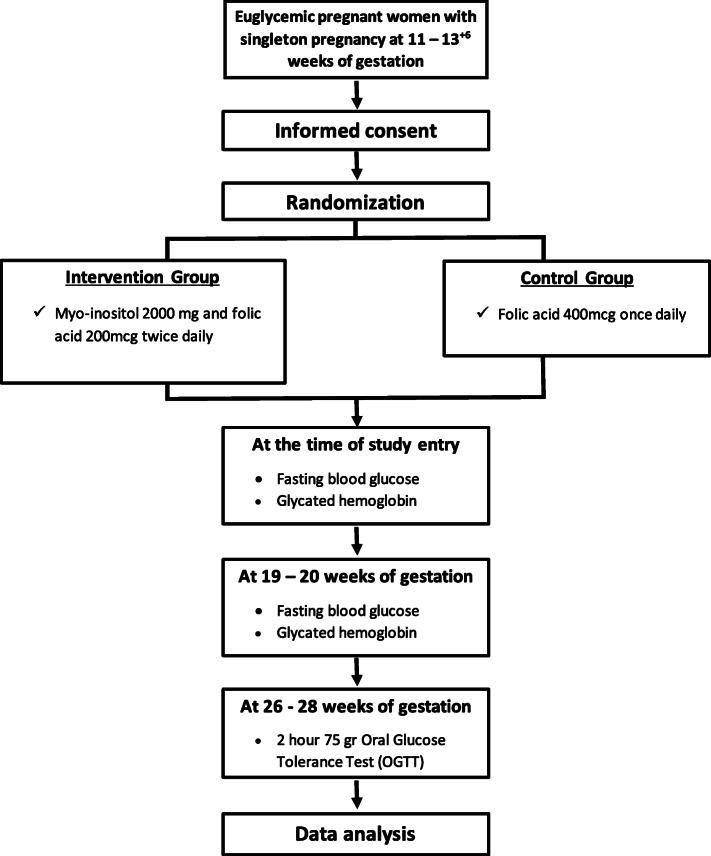
Trial protocol flow chart

This is a single-center, open-label, randomized controlled trial, which will be conducted in the First Department of Obstetrics and Gynecology in cooperation with the Diabetes Centre of First Department of Endocrinology in Alexandra Hospital in Athens, Greece. The study aims to enroll 160 healthy pregnant women over a 2-year period. Participants will be randomized, using a 1:1 ratio, to receive either myo-inositol and folic acid (4000 mg myo-inositol and 400 mcg folic acid daily) or folic acid alone (400 mcg folic acid daily). At 11–13^+6^ weeks of gestation and after providing written informed consent, patients will be randomly allocated in two groups until 26–28 weeks of gestation. At the time of study entry, Diabetes Centre of Alexandra Hospital will offer measurements of fasting blood glucose and glycated hemoglobin to all participants of both groups. At 19–20 weeks of gestation, the same measurements will be repeated to all participants. Finally, at 26–28 weeks of gestation, the 2 h 75 g oral glucose tolerance test (OGTT) will be offered for the diagnosis of gestational diabetes [4, 22]. The overall flow of the trial is shown in Fig. 1.

This trial was approved by the trial center’s ethics committee (Scientific Board of Alexandra Hospital, 28/06/2017, ref.: 520/22-06-2017) and is in accordance with the Declaration of Helsinki concerning Human and Animal Rights. Patient information will be treated confidentially with the use of encrypted individualized numbers. Neither the statistician involved nor the research physicians will have access to these data.

### Participants

Healthy adult pregnant women with singleton pregnancy at 11–13^+6^ weeks of gestation will be held eligible for inclusion in the present study.

Research assistants will discuss the expected outcomes and the potential adverse effects of myo-inositol in Greek or English language. Participants will be given a consent form and separate information sheets including information on the main aspects of the trial. Research assistants will obtain the signed consent form from patients willing to participate in the trial.

### Recruitment procedure

Participants will be informed and recruited at the scheduled appointment for the first trimester ultrasound at Maternal-Fetal Medicine Department of Alexandra Hospital for the measurement of nuchal translucency at 11–13^+6^ weeks of gestation.

Based on a 4-month pilot study, a recruitment rate of 20 patients eligible for the trial per month and 10 consenting patients per month is estimated, achieving adequate participant enrolment to reach the target sample size during the 2-year period of recruitment.

### Inclusion criteria

Patients will be enrolled if they are aged over 18 years old without pre-existing impaired glucose tolerance, have singleton pregnancy, and provide signed informed consent.

### Exclusion criteria

1. Age under 18 years
2. Multiple pregnancies
3. Pre-existing diabetes mellitus
4. Consumption of steroids
5. Hypertensive disorders
6. Hypothyroidism
7. Pre-existing renal or hepatic impairment
8. Beta thalassemia carriers
9. Vaginal bleeding (e.g., placental abruption)
10. Special diets (e.g., lactose intolerance)
11. Inadequate monitoring during pregnancy

### Interventions

As routine for the geographical area where this study is conducted, the control group will be given 400 mcg of folic acid orally per day, while the intervention group will be given 4000 mg of myo-inositol plus 400 mcg of folic acid per day (2000 mg myo-inositol and 200 mcg folic acid twice daily) from 11 to 13^+6^ weeks of gestation until 26–28 weeks of gestation. At the time of study entry, the Centre of Diabetes of Alexandra Hospital will offer measurements of fasting blood glucose and glycated hemoglobin to all participants of both groups. At 19–20 weeks of gestation, the same measurements will be repeated to all participants. Finally, at 26–28 weeks of gestation, the 2 h 75 g oral glucose tolerance test (OGTT) will be offered for the diagnosis of gestational diabetes [4, 22]. An evaluation of maternal glycemic status will be performed on monthly intervals until delivery and neonates will be followed up until 6 months of life.

Adherence will be monitored at every post-allocation visit by the responsible research physician. The participants must return the bottles at each visit and the physician will count the tablets left in the bottles. Participants with inadequate adherence will be recorded.

The oral combination of myo-inositol and folic acid and the oral tablet of folic acid alone are manufactured by Italfarmaco S.p.A (Milan, Italy).

**Fig. 2:**
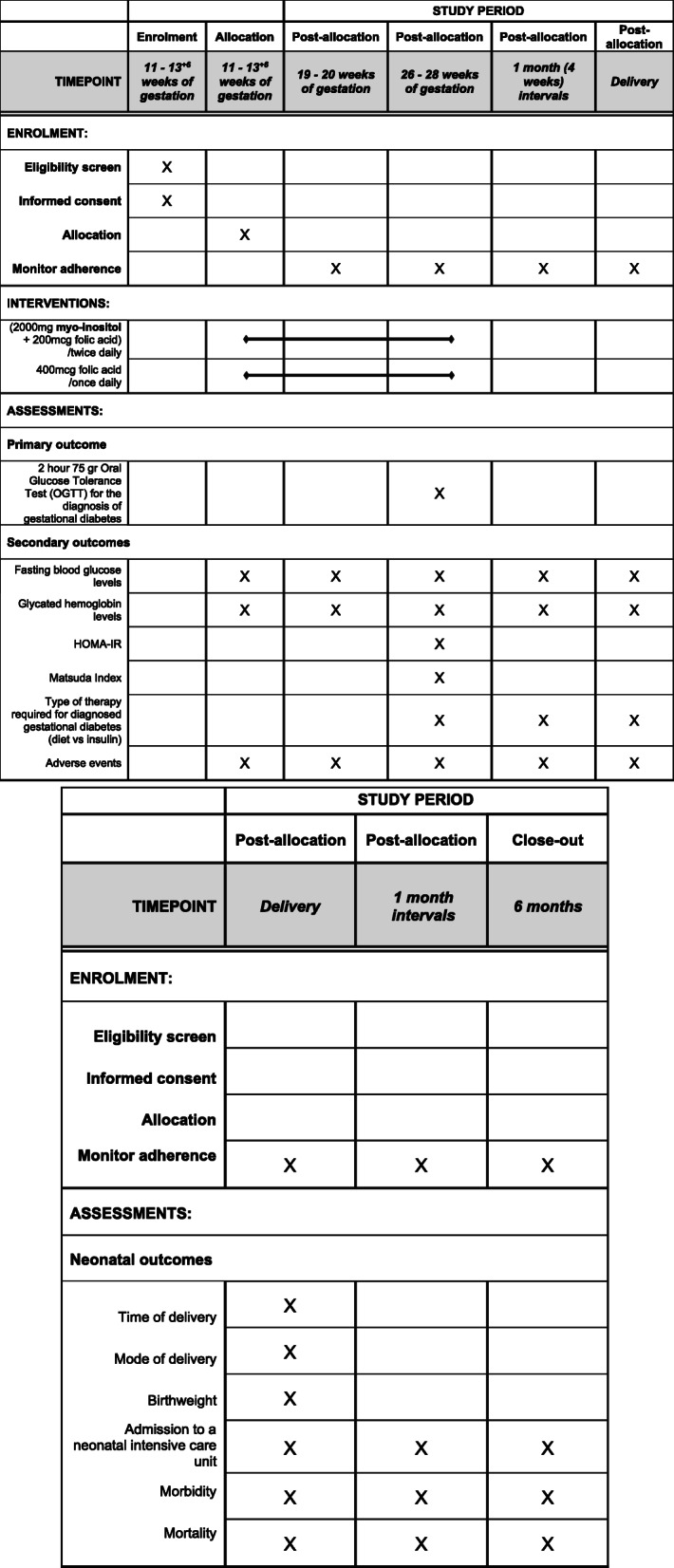
Time schedule of enrolment, interventions, and assessments

A timeline of the study procedures is presented in Fig. 2.

### Randomization and blinding

Allocation sequence will be based on computer-generated randomization that will be organized by an independent research assistant. Allocation concealment will be ensured with the use of sequentially numbered opaque sealed envelopes which will be constructed and secured by an independent research assistant. Complete blinding of the procedure will not be possible, due to limitations of funding which is provided by the investigators only; hence, placebo will not be similar to the administered regimen (myo-inositol). We do acknowledge the limitation of the non-blinding process in our study; however, given the fact that reported outcomes will not be subjective (physician or patient reported) but rather based on actual biochemical data (OGTT), we believe that the lack of blinding will not affect the integrity of our findings. However, both the statistician and the research physicians will be blind to the selection of participants as this will be performed by an independent research assistant.

### Outcome measures

#### Primary outcomes

The primary outcome measure in the trial is gestational diabetes incidence rate at 26–28 weeks of gestation. According to the results of a 75 g oral glucose tolerance test (OGTT) at 26–28 weeks of gestation, gestational diabetes would be diagnosed if one or more values met or exceeded the following levels of glucose: fasting 5.1 mmol/l (92 mg/dl), 1 h post glucose 10.0 mmol/l (180 mg/dl), and 2 h post glucose 8.5 mmol/l (153 mg/dl) [4, 22].

#### Secondary outcomes

Secondary outcome measures include fasting blood glucose levels; glycated hemoglobin levels; insulin resistance level, evaluated by homeostasis model assessment of insulin resistance (HOMA-IR) and Matsuda Index; incidence rate of diet-treated gestational diabetes; and diabetes requiring insulin therapy.

$$ {\displaystyle \begin{array}{l}\mathrm{HOMA}\hbox{-} \mathrm{IR}=\mathrm{Fasting}\ \mathrm{glucose}\ \left(\mathrm{mmol}/\mathrm{L}\right)\times \mathrm{Fasting}\ \mathrm{insulin}\ \left(\mathrm{mIU}/\mathrm{L}\right)/\kern0.5em 22.5\kern0.5em \mathrm{or}\\ {}\mathrm{HOMA}\hbox{-} \mathrm{IR}=\mathrm{Fasting}\ \mathrm{glucose}\ \left(\mathrm{mg}/\mathrm{dL}\right)\times \mathrm{Fasting}\ \mathrm{insulin}\ \left(\mathrm{mIU}/\mathrm{L}\right)/405\end{array}} $$

$$ \mathrm{MATSUDA}-\mathrm{I}={10}^4/\surd \left(\mathrm{Fasting}\ \mathrm{glucose}\times \mathrm{Fasting}\ \mathrm{insulin}\right)\times \left(\mathrm{Mean}\ \mathrm{glucose}\times \mathrm{Mean}\ \mathrm{insulin}\right) $$

Maternal hyperglycemia and hyperinsulinemia will be assessed in monthly follow-up visits until delivery as well to determine the impact of myo-inositol on the overall pregnancy course.

Neonatal outcomes (time and mode of delivery, birth weight, admission to a neonatal intensive care unit, perinatal morbidity, perinatal mortality) will be reported as a secondary analysis of our findings in an upcoming paper, following close follow up of neonates up until the age of 6 months.

### Safety assessments

Inositol and folic acid are considered supplements and are not expected to be associated with adverse events when administered at the normal daily dosage. However, participants will be asked to report all potential adverse events (AEs) at each visit point, and all AEs reports will be recorded and assessed by the investigators. In the case of unexpected serious adverse effect, the participant will be hospitalized or treated at the high-risk pregnancy department of our hospital at no charge.

A data monitoring committee will not be formed as myo-inositol is a natural product that is daily consumed in various compounds such as fruits and grains. Furthermore, previous trials that evaluated inositol in pregnancy have not reported adverse effects. Nevertheless, an interim analysis will be performed following the recruitment of the first 40 patients to assess for potential unexpected adverse effects that will be considered as a reason to discontinue the trial.

### Sample size calculation

Given the lack of previous data in this field, we sought to determine the sample size of our study based on two types of calculations. The largest sample size was finally used to ensure that our study would not be underpowered. The level of statistical significance was set at 0.05 and the power of the study at 0.80 using an 1/1 allocation. In our first attempt, we used the study of Genazzani et al. [10] and calculated the sample size taking into account the fasting insulin levels. The effect size was calculated to be 0.86 and the sample size in each group of 30 women. In the second attempt, we used the decline in the prevalence of GDM that was observed in the study of D’Anna et al. [20] (14 vs. 33.6%). According to the latter sample size calculation, 73 women were needed in each group. With an estimated 10% of patient withdrawal and lost to follow up rates, the final number of enrolled women was estimated to 160 women. Given the relevance of the prevalence of GDM in the latter study to that of the Greek population that is treated in our hospital [23] and other studies in this field that supports an almost 50% reduction in GDM following inositol treatment [16, 24], we chose to include the latter calculation in our study. The G power tool v. 3.1.9.2 was used in all cases.

### Data management and quality control

The main study will be preceded by a 4-month pilot study to assess the compliance of participants in the study group related to the consumption of myo-inositol and their compliance regarding the re-evaluation of glucose levels at 19–20 and 26–28 weeks.

All data will be tabulated in preconstructed tables using the Microsoft Access software, which ensures the homogeneity of data entry. All electronic data will be double-checked from two independent researchers to preclude the possibility of misreporting. Only predefined variables will be enlisted. Interim evaluation of the process of data handling will be provided following the recruitment of the first 40 patients to audit patient adherence as well as the data acquisition process. The access to the study’s dataset (for both interim evaluation and final analysis) will be granted only to the statistician that will be involved in the study. Physicians will not be able to access this dataset prior to the publication of this study.

### Statistical analysis

All analyses will be performed in the intention to treat (ITT) population of our study. Sensitivity (per-protocol analysis) will be also provided taking in mind parameters that may deviate from planned treatment such as failure to adhere to the recommended dosage, drug dose change requested by the primary physician or the patient in response to adverse effects, and concurrent consumption of insulin. The normality of the distribution of our findings will be assessed with the Kolmogorov-Smirnov test and graphical methods. Parametric and non-parametric tests will be used based on these latter findings accordingly. The chi-square test will be used to compare discrete variables and Fisher’s exact test will be used to compare discrete variables with less than 5 observations (if any). Adjusted analysis will be performed as well taking into account the variables of age and body mass index. Logistic regression will be performed in an attempt to identify the impact of myo-inositol in a multivariate model analysis (sensitivity analysis). Complete case analysis will be performed and multiple imputation will not be attempted. Exploratory subgroup analyses will be performed for subsets of patients with risk factors (obesity, history of GDM, family history of diabetes, PCOS) if an arbitrary cut-off of at least 20 patients per group (myoinositol – control) will be reached. All analyses will be performed with the SPSS 21.0 package (IBM Corp. Released 2012. IBM SPSS Statistics for Windows, Version 21.0. Armonk, NY: IBM Corp.).

## Discussion

Pregnancy is characterized by insulin resistance and consequent hyperglycemia, which may have evolved in order to ensure that the fetus will have a continued supply of nutrients even in times of famine. The estimated global prevalence of hyperglycemia in pregnancy is 16.9% [25]. During pregnancy, crucial alterations in the mother’s metabolism take place in an attempt to support fetal development. During the second half of pregnancy, insulin resistance is characterized by severely high levels and insulin secretion increases approximately by 200 to 250%, in order to maintain euglycemic status [26]. If insulin secretion is insufficient, hyperglycemia and consequent GDM will develop.

The prevention of GDM is of vital importance because hyperglycemia is associated with teratogenic effects on fetal anatomy and development, resulting in severe long-term adverse effects on the offspring. Neonates have increased risk of macrosomia and birth defects [27]. They also have increased risk to develop childhood obesity, glucose intolerance in early adulthood [28, 29], hypertension, and gestational diabetes later in their lives [30]. Therefore, glucose control during pregnancy is crucial.

Myo-inositol has been widely studied for its role as an insulin-sensitizing factor. The biochemical mechanism through which myo-inositol improves the metabolic state of patients with GDM and other insulin-resistant conditions remains unknown. One hypothesis refers to the direct intracellular action of myo-inositol on the activation of acetyl CoA carboxylase, which stimulates lipogenesis [31]. Another hypothesis refers to the role of myo-inositol as a precursor of D-chiro-inositol containing inositol phosphoglycan, which is bound to the extracellular matrix of the cells. The binding of insulin to specific receptors stimulates D-chiro-inositol transfer intracellularly [32], explaining its mediating role in the insulin-signaling pathway [29].

Several studies have demonstrated the insulin-sensitizing role of myo-inositol in conditions such as PCOS, post-menopausal metabolic syndrome, type 2 diabetes, and GDM. PCOS is a widely studied syndrome associated with insulin resistance, for which insulin-sensitizing factors have been proposed as putative treatments to improve the hyperinsulinemia-related dysfunction of ovarian response to endogenous gonadotropins. Among the insulin-sensitizing compounds, myo-inositol has shown a beneficial effect on improving insulin sensitivity and restoring spontaneous ovarian activity, and consequently fertility in PCOS patients [10, 11, 29, 33, 34]. Post-menopausal metabolic syndrome is another insulin-resistant syndrome with a good response to myo-inositol administration. In fact, it has been demonstrated that myo-inositol may decrease insulin resistance by approximately 70% in post-menopausal women with metabolic syndrome [19, 35]. Another common disorder in which insulin resistance constitutes a crucial factor is type 2 diabetes mellitus (T2DM). T2DM is characterized by an increase in insulin resistance of the target tissues and a deficiency of insulin secretion from the pancreas [36]. The first-line therapy for T2DM is metformin, an insulin sensitizer drug [37, 38]. However, myo-inositol and D-chiro-inositol have shown a direct beneficial effect on glycemic parameters of patients with T2DM, such as a significant reduction in blood glucose and HbA1c levels. Furthermore, inositol administration did not lead to any side effect, confirming the safety of this intervention [39].

Several studies reported an active role of oral myo-inositol in insulin resistance of GDM patients. In a randomized trial, Corrado et al. [14] found a greater decrease of insulin resistance in third-trimester GDM pregnancies treated with myo-inositol and folic acid than in a control group of third-trimester GDM pregnancies with folic acid alone (homeostasis model assessment of insulin resistance *P* = 0.0001).

In a retrospective study conducted in pregnant women affected by PCOS, D’Anna et al. [18] observed that myo-inositol administration, throughout the pregnancy course, may reduce the prevalence of GDM. In particular, these authors demonstrated a lower prevalence of GDM in PCOS women who achieved conception on myo-inositol supplement and continued this regimen during pregnancy, compared with PCOS women who conceived on metformin and discontinued it after the diagnosis of pregnancy (17.4 vs. 54%, *P* = 0,001).

Another open-label RCT [13] reported a significant decrease of GDM incidence in non-obese women, with a family history of T2D, treated with myo-inositol and folic acid from the end of the first trimester throughout the remainder of the pregnancy compared with similar control subjects treated with folic acid alone (6 vs. 15.3%, *P* = 0.04). Moreover, fetal macrosomia was significantly reduced (0 vs. 7%, *P* = 0.007) in the myo-inositol group and the mean birth weight was also significantly reduced in the treatment group (*P* = 0.018). The authors also reported a statistically significant decrease of fasting plasma glucose (*P* = 0.001) and 1-h plasma glucose on the 75 g oral glucose tolerance test (OGTT) (*P* = 0.02) in the myo-inositol group compared to the control group. The same authors [20] reported a lower incidence of GDM (14 vs. 33.6%, *P* = 0.001) and a greater reduction in the homeostasis model assessment of insulin resistance (− 1.0 ± 3.1 vs. 0.1 ± 1.8, *P* = 0.048) among 99 obese pregnant women treated with myo-inositol and folic acid from the first trimester to delivery compared to 107 obese pregnant women treated with folic acid alone.

Matarelli et al. [16] demonstrated that maternal/fetal/neonatal GDM in women at high risk was well controlled when treated with myo-inositol. In particular, women treated with myo-inositol showed a significantly lower prevalence of GDM in mid-pregnancy (risk ratio (RR) = 0.127, 95% CI [0.032–0.502], *P* = 0,001), required less insulin therapy (RR = 0.136, 95% CI [0.018–1.031], *P* = 0.053), delivered at a later gestational age (95% CI [− 2.578 to − 0.948], *P* = 0.001), and had significantly smaller babies (birth weight is expressed as percentiles, 95% CI [10.807–30.116], *P* = 0.001) with fewer episodes of neonatal hypoglycemia (RR = 0.052, 95% CI [0.003–0.849], *P* = 0.038) compared to placebo.

A recent study conducted by Dell’Edera at al [40]. demonstrated that the supplementation with D-chiro-inositol and D-myo-inositol allows a better control of maternal glycemia resulting in a lower incidence of GDM (*P* = 0.0028) and more favorable perinatal outcomes referring to the risk of macrosomia (*P* = 0.0099).

Similar findings were confirmed in a recent animal study, which demonstrated that myo-inositol supplementation is associated with adipose tissue markers of improved insulin sensitivity and glucose uptake in a mouse model of GDM [41].

Most RCTs concluded that more studies are necessary to further evaluate the safety and clinical effectiveness of myo-inositol supplementation during pregnancy. A recent review of Cochrane Database [42] highlighted the need for further studies with participants of varying ethnicities and with varying risk factors for GDM.

This randomized controlled trial is designed to evaluate the efficacy of myo-inositol supplementation from the first trimester of pregnancy to the time of GDM diagnosis in reducing the incidence of GDM. Results of this study will provide evidence regarding the value of myo-inositol as a safe intervention for the prevention of gestational diabetes.

## Trial status

Protocol version number and date: 520/22-06-2017

Recruitment start date: 03/12/2019

Recruitment end date: 01/01/2022

Currently, the number of recruited patients is 48 for the first 6 months overcoming the desired number (42) for the same period of time. Therefore, the current status of the recruitment into the trial demonstrates that the recruitment goals can be reached.

## Dissemination policy

The results of the study will be submitted for publication to peer-reviewed medical journals and presented at symposia regardless of the outcomes.

## Supplementary information

**Additional file 1.** SPIRIT 2013 Checklist: Recommended items to address in a clinical trial protocol and related documents.

## Data Availability

Not applicable.

## Abbreviations

AEs: Adverse events
BMI: Body mass index
GDM: Gestational diabetes mellitus
HOMA-IR: Homeostasis model assessment of insulin resistance
ITT: Intention to treat
OGTT: 2 h 75 g oral glucose tolerance test
PCOS: Polycystic ovary syndrome
T2D: Type 2 diabetes
